# Wavelength stability in a hybrid photonic crystal laser through controlled nonlinear absorptive heating in the reflector

**DOI:** 10.1038/s41377-018-0043-8

**Published:** 2018-07-25

**Authors:** Andrei P. Bakoz, Alexandros A. Liles, Alfredo A. Gonzalez-Fernandez, Tatiana Habruseva, Changyu Hu, Evgeny A. Viktorov, Stephen P. Hegarty, Liam O’Faolain

**Affiliations:** 1 Centre for Advanced Photonics and Process Analysis & Department of Physical Sciences, Cork Institute of Technology, Cork, T12P928 Ireland; 2 Tyndall National Institute, University College Cork, Lee Maltings, Dyke Parade, Cork, T12R5CP Ireland; 3 School of Physics & Astronomy, University of St Andrews, North Haugh, St Andrews, KY16 9SS UK; 4 National Research University of Information Technologies, Mechanics and Optics, Saint Petersburg, 199034 Russia; 5 Present Address: IMEC, Department of Information Technology (INTEC), Photonics Research Group, Ghent University, Technologiepark-Zwijnaarde 15, 9052 Ghent, Belgium; 6 Present Address: National Institute of Astrophysics, Optics and Electronics, 72840 Tonantzintla, Mexico

## Abstract

The need for miniaturized, fully integrated semiconductor lasers has stimulated significant research efforts into realizing unconventional configurations that can meet the performance requirements of a large spectrum of applications, ranging from communication systems to sensing. We demonstrate a hybrid, silicon  photonics-compatible photonic crystal (PhC) laser architecture that can be used to implement cost-effective, high-capacity light sources, with high side-mode suppression ratio and milliwatt output output powers. The emitted wavelength is set and controlled by a silicon PhC cavity-based reflective filter with the gain provided by a III–V-based reflective semiconductor optical amplifier (RSOA). The high power density in the laser cavity results in a significant enhancement of the nonlinear absorption in silicon in the high *Q*-factor PhC resonator. The heat generated in this manner creates a tuning effect in the wavelength-selective element, which can be used to offset external temperature fluctuations without the use of active cooling. Our approach is fully compatible with existing fabrication and integration technologies, providing a practical route to integrated lasing in wavelength-sensitive schemes.

## Photonic crystals: temperature stable lasers

Silicon photonic crystal technology has enabled the creation of highly compact, uncooled lasers that can be wavelength stabilized over a wide range of temperature. The lasers, designed and fabricated by researchers from Ireland, Scotland, and Russia could be ideal for use in high-capacity optical interconnects in datacenters. The lasers consist of two parts: a ridge-waveguide AlGaInAs/InP semiconductor optical amplifier to provide gain; and a silicon photonic crystal resonant reflector. As the drive current to the laser is increased, the absorption of intracavity light within the silicon photonic crystal leads to a wavelength shift in laser operation. By using this shift to counteract any wavelength changes due to ambient temperature variations, the researchers demonstrated that the laser’s wavelength can be stabilized without active cooling to within ±0.38 nm over a 20–80 °C temperature range.

## Introduction

Silicon photonics takes advantage of the mature complementary metal-oxide semiconductor (CMOS) infrastructure and processes and is actively pursued for the implementation of complex optical components and photonic integrated circuits (PICs) at low cost and high volumes. Despite constant refinement of silicon photonics technology to meet the evolving requirements for applications, the poor light emission ability of silicon remains a constraint. As a result, the most essential building block of an optical system, an efficient light emitter, remains absent in PICs based on silicon.

Chip-scale lasers are key elements for many applications, ranging from data communications, especially wavelength-division multiplexing (WDM) systems, to various optical sensing applications, such as trace-gas detection. A commonly used method for circumventing the above problem is the combination of III–V materials with silicon via heterogeneous or hybrid integration. Based on such schemes, a number of different laser configurations have been proposed and demonstrated^1–3^. However, uncooled operation is a prerequisite for cost-sensitive applications, but many lasers integrated on silicon still struggle to operate at temperatures above 50 °C. Furthermore, precise wavelength control over a range of ambient temperatures is a requirement for WDM and optical sensing systems, which is a fundamental problem for uncooled operation.

In this article, we demonstrate a hybrid laser architecture that comprises a III–V gain element and a silicon photonic crystal (PhC) cavity-based resonant reflector. The unique features of PhC cavities are exploited to demonstrate a new type of laser that is capable of producing output powers of several milliwatts. In this configuration, the high intra-laser cavity power (10 s of milliwatts) results in unprecedented stored energy densities in the PhC resonator. The enhanced light–matter interaction in the high-quality-factor (*Q*), low-mode-volume (*V*) PhC cavity leads to an enhancement of the nonlinearities in silicon, rendering its resonance a strong function of the output power—an effect that we use to realize an athermal lasing wavelength response during uncooled operation.

## Materials and methods

A 250-μm ridge waveguide AlGaInAs/InP reflective semiconductor optical amplifier (RSOA) was utilized as the gain chip. The Al^3+^ ions contained in the AlGaInAs quaternary quantum wells deepen the potential well and obstruct carrier leakage at elevated temperatures, thus facilitating higher temperature operation^4^. The back facet of the RSOA was high-reflection coated with >90% power reflectance, and the front facet was anti-reflection (AR) coated to minimize back-reflections. The RSOA die was mounted onto an aluminum nitride ceramic tile with its front end protruding off the sub-mount. The silicon-based reflector chip consisted of a low-refractive-index waveguide vertically coupled to an oxide-clad PhC cavity. The dispersion adapted (DA) PhC cavity design was specifically chosen here due to its suitability for mass manufacture via deep ultra-violet photolithography^5^. The PhC cavity was fabricated on a standard 220-nm silicon-on-insulator (SOI) platform by electron-beam lithography and reactive ion etching, similar to ref. ^6^. Accuglass-T by Honeywell was used for the oxide upper cladding, resulting in a multi-layer structure widely used in PICs^7,8^. SU-8 polymer (*n* ~ 1.58 at 1550 nm) was selected for the waveguide, but the possibility of using other low-index materials has also been demonstrated^6^. The width and height of the waveguide were ~3.1 and ~2.1 μm, respectively, and its facets were normal to the SOI-chip surface and normal-incidence AR coated with a single MgF_2_ layer. Both the SU-8 and RSOA waveguides operated on a  single transverse mode in all regions.

**Fig. 1:**
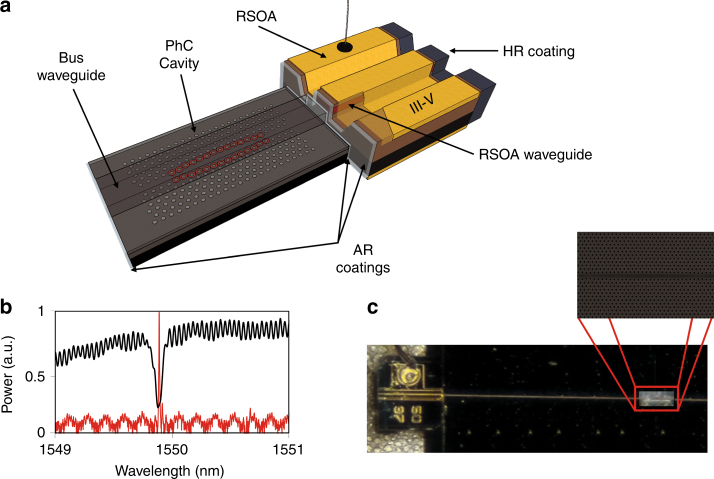
The hybrid PhC laser configuration comprising an RSOA and a Si PhC-based resonant mirror. A schematic representation of the device is given in (**a**), and a microscope view is shown in (**c**). The utilized reflector consists of a low-index dielectric waveguide located vertically over a PhC cavity on silicon-on-insulator (SOI), with the two parts separated by a thin buffer layer of oxide that enables their evanescent coupling^6^. The laser cavity is formed by butt-coupling the RSOA to the waveguide on the silicon chip, which acts as a narrowband reflector at the resonant wavelengths of the PhC cavity, as described in ref. ^9^. The filter reflection spectrum (black curve) and laser spectrum (red curve) are  superimposed in (**b**)

A conceptual representation of the considered laser architecture is shown in Fig. 1. The laser cavity is formed by butt-coupling the RSOA waveguide to one end of the SU-8 waveguide on the reflector chip, as shown in Fig. 1a. The low refractive index of the bus waveguide combined with its large cross-section enables better matching with the mode of the RSOA, resulting in low butt-coupling losses (<1.5 dB) and improvements in the tolerance to misalignment between the two parts. Experiments for continuous-wave (CW) lasing characterization were carried out by mounting the RSOA carrier onto a five-axis alignment stage and the silicon reflector chip onto a single-axis horizontal translation stage. Alignment optimization between the RSOA and the silicon reflector chip was carried out below the laser threshold, with an infrared InGaAs camera equipped with a 100× objective lens used for observation from above. Both the RSOA and the reflector chips could be independently temperature controlled by Peltier elements, within the temperature range of 20–80 °C. It should be noted that the Peltier elements were used for the sole purpose of studying the lasing characteristics of the device at varying temperature; the device runs effectively without active cooling. The laser output was collected at the other end of the SU-8 waveguide with a lensed optical fiber mounted on a three-axis stage. A fiber coupled isolator with isolation >50 dB was placed after the lensed fiber to minimize undesired reflections. A 2 × 2 fiber coupler was used for the simultaneous observation of the lasing spectra and output power.

## Results and discussion

During operation, the light generated in the RSOA is coupled to the SU-8 polymer waveguide. At the resonant wavelength of the PhC cavity, light couples evanescently from the waveguide mode to the PhC cavity mode. Once power is built up inside the PhC cavity, light couples back to the bus waveguide in two different directions: forwards—to the output facet of the waveguide—and backwards—to the RSOA. The backward-propagating light component acts as wavelength-selective feedback, with a linewidth on the order of 0.01–0.1 nm (depending on the selected coupling conditions), resulting in the formation of a laser cavity between the reflective facet of the RSOA and the PhC cavity (Fig. 1a, c). The emitted wavelength is determined by the longitudinal mode of the laser cavity that lies within the PhC cavity reflection band (Fig. 1b).

In general, due to their large quality-factor–modal volume (*Q*/*V*) ratio, PhC cavities can be used to realize extraordinarily high stored energy densities. As a consequence, in the case of silicon PhC cavities, nonlinear phenomena can be observed even at low (on the order of μWatt) input powers^10^. In particular, two photon absorption (TPA) scales with the square of the energy stored in a silicon cavity^11,12^. Every pair of TPA-absorbed photons gives rise to an electron–hole pair that may result in the absorption of further photons via the free carrier absorption (FCA) mechanism.

Thus, in the examined situation, the power decay rate of the silicon PhC cavity on the reflector chip (defined as Γ = *ω*_0_/2*Q*_L_, with *ω*_0_ being the resonant frequency and *Q*_L_ the loaded quality factor of the cavity), consists of four components: decay due to radiation losses (described by Γ_rad_), decay into the bus waveguide mode (Γ_coup_), decay due to TPA (Γ_TPA_), and decay due to FCA (Γ_FCA_), which is proportional to the free carrier density in the volume of the optical mode. Based on the above discussion, the absorbed optical power in the modal volume of a PhC cavity is given by

1 $$p = h\nu \left( {\Gamma _{\mathrm{TPA}} + \Gamma _{\mathrm{FCA}}} \right)$$

The recombination of the free carriers generated by the aforementioned nonlinear absorption ultimately results in heat dissipation in the PhC cavity. The region in which heat is generated very closely matches the spatial distribution of the optical mode (with carrier diffusion and thermal diffusion slightly enlarging this region), automatically constituting one of the most efficient localized heating mechanisms possible.

As the PhC cavity temperature sets the resonant wavelength, and hence the emitted wavelength in the studied laser architecture, we propose tuning of the latter through the laser output power—a technique we refer to as power tuning. More specifically, we suggest that wavelength stability in the examined laser configuration can be realized for uncooled operation by balancing the variation in the ambient temperature with changes in the PhC cavity temperature through heating caused by carrier recombination. If the laser power is appropriately decreased as higher operating temperatures are reached, the two effects can be adjusted to cancel each other out, giving a zero net shift of the emitted wavelength. For a fixed laser output power, the ratio between the absorptive decay rate and the total decay rate determines the amount of power dissipated as heat in the cavity. Γ_coup_ can be controlled by the separation between the bus waveguide and the PhC cavity, whereas the other terms are fundamental or technological constants. In this initial experiment, we chose Γ_coup_ so that the dissipated power is on the order of a few milliwatts.

**Fig. 2:**
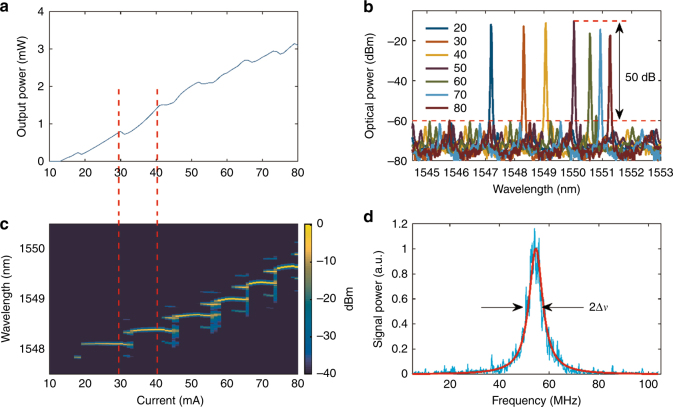
Laser characterization. **a** The  light-current (LI) curve without temperature stabilization and (**b**) single-mode (SM) lasing at 80 mA for a temperature range of 20–80 °C, without power tuning. **c** False color plot of the time-averaged optical spectrum as a function of the upswept drive current, with the *x* axis matched to that of (**a**). From the change in the lasing wavelength, we can deduce an increase in PhC temperature of 15 °C. **d** Delayed self-heterodyne measurement of the laser linewidth in a single-mode region. The central frequency of the acousto-optic modulator (AOM) was 55 MHz; the measured linewidth Δ*ν* = 4.5 MHz

Following the process described in the Materials and Methods section, the CW characteristics of multiple lasers with the proposed architecture were measured at room temperature, without active cooling, for driving currents up to 100 mA. Single longitudinal mode lasing was realized by aligning a longitudinal mode of the laser cavity with the reflector band. Typical laser threshold currents were found to be in the 10–20 mA range. Output powers of several mW were measured in every case, with a maximum waveguide-coupled wall-plug efficiency of 8% at 40 mA. Figure 2 shows the data obtained for an indicative device. The measured side-mode suppression ratio was determined to be in excess of 40 dB over the full current range considered, with a maximum value of 50 dB. The single-mode laser linewidth above the threshold was determined to be 4.5 MHz by a delayed self-heterodyne linewidth measurement. Figure 2a shows the light-current (LI) curve for the laser under consideration, which exhibits a series of kinks, attributed to mode hopping. It should be noted that the mode hopping occurs due to transitions between adjacent longitudinal modes of the laser cavity and is not associated with transitions between different modes of the PhC cavity, the FSR of which (~8 nm^6,13^) is much larger than the observed hops.

As the driving current was swept from the threshold to 80 mA (without active cooling), the resonant wavelength of the PhC reflective filter was nonlinearly redshifted by ~3 nm due to absorptive heating, which corresponds to multiple longitudinal mode spacings of the laser cavity. The transitions between different longitudinal modes can also be seen in the time-averaged optical spectra (Fig. 2c) for the device under test. The existence of two modes with weak sidebands observed at the transition is due to the averaging of the temporally unstable longitudinal mode hopping.

**Fig. 3:**
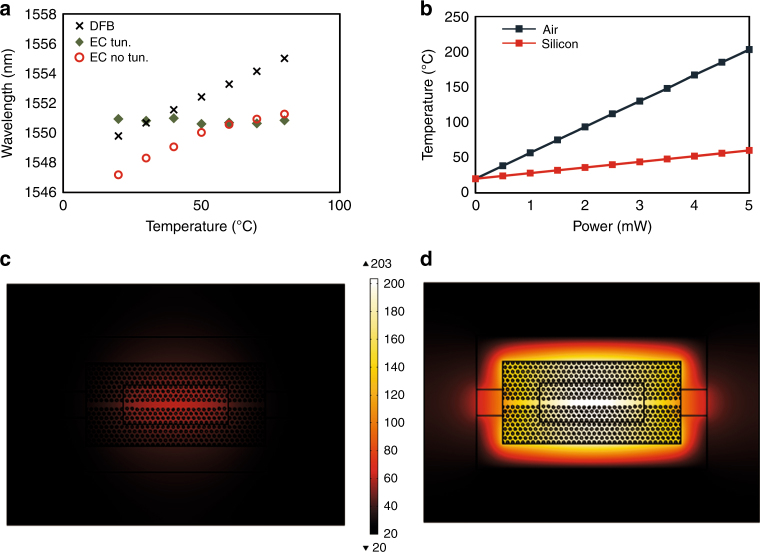
Temperature stable operation **a** Emitted wavelength as a function of temperature for a packaged DFB laser at a drive current of 80 mA (black crosses), a PhC laser at 80 mA without power tuning (red circles), and the same PhC laser with power tuning realized by drive current tuning (from 150 to 50 mA, for 20–80 °C, respectively—green rhombi). **b** Simulated temperature as a function of dissipated power for the SOI PhC (red) and the undercut PhC (dark blue). **c** Thermal profile of the PhC cavity on the SOI resonant reflector chip for a dissipated power of 2 mW. **d** Thermal profile of an undercut PhC cavity for a dissipated power of 2 mW

To put the performance of the power-tuned hybrid laser into perspective, we compare its wavelength response to changes in ambient temperature with that of a distributed feedback (DFB) laser, which is the most commonly utilized light source for applications that require a high spectral purity. For this comparison, the temperature of both the RSOA and the silicon chip was reduced from 80 to 20 °C in steps of 10 °C via Peltier elements. As the temperature of the substrates was decreased, the blueshift in the reflection peak was compensated without active cooling by increasing the drive current, which led to an increase in the absorptive heating in the PhC cavity. In this way, a variation of only ±0.38 nm around the central lasing wavelength (*λ*_0_ = 1550.85 nm) was realized over the entire considered temperature range. As a reference, we examined the change in the emitted wavelength of a JDS Uniphase CFQ935 DFB laser as a function of the substrate temperature. A shift of ~6 nm was measured for the aforementioned 60 °C variation—more than an order of magnitude larger than in the power-tuned PhC laser. The observed change in wavelength in the DFB laser is a result of the effect of temperature on the grating, which determines the lasing frequency through the Bragg condition, and is related to the temperature sensitivity of the grating material. The comparative results are summarized in Fig. 3a.

To obtain a further understanding of the power tuning mechanism in the PhC laser configuration, the temperature of the PhC as a function of dissipated optical power was modeled, as shown in red in Fig. 3b. For the same conditions as in the experiments reported here, our simulations revealed a temperature rise of 10 °C per milliwatt of dissipated power. Indicatively, Fig. 3c shows the thermal profile of a DA PhC cavity on SOI for a dissipated power of 2 mW in it. As explained previously, the absorptive heat is generated in the 0.495 μm^3^ volume of the mode of the PhC cavity, which very effectively raises the temperature of the resonator. This heating mechanism is more than twice as efficient as a conventional metal micro-heater^14^, which has to be located at some distance (>1 μm) from the optical mode to avoid optical absorption losses. Such a high heating efficiency enables strong variations of the PhC cavity temperature as a function of coupled power and, by extension, of the intra-cavity power of the laser. In our experiments, stability of the emitted wavelength over a 60 °C span of substrate temperature (from 20 to 80 °C) was realized by a 2.3 mW change in the output power, corresponding to an approximately 6.5 mW change in the power dissipated in the PhC cavity. This variation in laser output as the temperature changes implies a requirement for a receiver to be able to handle a variation in the average power level. The power variations as a result of power tuning in the device considered here are well within the range of modern optical receivers.

The power-tuning technique relies on nonlinear absorption-generated heat in silicon combined with increased light–matter interaction (corresponding to exceptionally high power density) in the wavelength-selective element of the laser cavity. As a result, silicon PhC cavities, offering the ultimate *Q*/*V* ratio, can exhibit a power-tuning performance superior to that of other systems. For example, ohmic heating in the reflective grating can result in changes in the lasing wavelength of a DFB laser as the drive current is varied. However, as for a given pump power, the energy densities in Bragg gratings are much lower than those in PhC cavities, the dependence of the emitted wavelength of such a laser on the output power is much weaker (and typically linear), implying that an impractically large change in drive current would be required to realize stability via power tuning over the considered 20–80 °C range. Indicatively, an increase in pump current from 50 to 200 mA was necessary in the above CFQ935 DFB laser to compensate for a substrate temperature change of only 10 °C. Another interesting scheme for silicon-compatible single-mode lasing sources involves the recently demonstrated hybrid III–V/silicon ring resonator lasers^15^. Ring resonators can provide significant field enhancement, and similar to PhC cavities, nonlinear optical transmission and bistability have been reported in these devices^16^. However, the mode volume of a typical ring resonator is ten times larger than that of a PhC cavity (~5.6 μm^3^ for a ring resonator with a radius of 10 μm, following ref. ^17^, compared to ~0.495 μm^3^ for the PhC cavity used here), and thus the relative field enhancement is much smaller. As two-photon absorption is proportional to the square of the light intensity, for the same *Q*-factor and input power, the TPA rate in such a ring resonator will be a factor of 100 less than that in the PhC. Coupled with the larger heat capacity, the dependence of the output wavelength on power for a ring resonator hybrid laser will be very weak, with the potential for power tuning being insignificant.

Toward further reduction of the requirements on the dissipated power and the output power variation for the realization of power tuning in the examined hybrid PhC laser architecture, we consider a situation in which the silicon substrate is removed from underneath the PhC cavity by, for example, xenon difluoride (XeF_2_) etching^17^. The dark blue curve in Fig. 3b shows the simulated temperature variation as a function of power dissipated in the cavity for such a case. As the thermal isolation is now higher, the heating of the PhC cavity is even more efficient, giving a calculated change of 33 °C/mW, compared to the 10 °C/mW change in the case of a reflector based on a silicon substrate. In this new scenario, the coupling between the waveguide and the PhC cavity ($\Gamma _{\mathrm{coup}}$) must be re-optimized to reflect this increased sensitivity. As a result, the heating power range required for power tuning over the same substrate temperature span (80–20 °C) can be reduced to ~2 mW, with the slope efficiency of the laser improved by virtue of the lower reflectivity of the PhC reflector.

Lasers incorporating the architecture proposed here are compatible with mass production techniques. While the authors prefer the hybrid integration vision of ref. ^18^, our wavelength stabilization technique is equally applicable to other integration schemes, for example, the heterogeneous integration approach of refs. ^1,19^. This technique simply requires a high *Q*/*V* optical resonator (e.g., a PhC cavity), which can be well realized in these technologies. Chip bonding^4^ and transfer printing^20^ are particularly appropriate to realize a hybrid silicon PhC–polymer–RSOA scheme. The simulated mode area of the polymer waveguide is 4.5 µm^2^, which is a close match to the mode area of 4.6 µm^2^ for the RSOA used here, with slight differences in the shape. Such mode areas give a good tolerance to misalignments, resulting in a calculated reduction of 15% in the coupling efficiency and an experimentally measured 5 mA increase in threshold current, for a 500 nm misalignment. The aforementioned sub-micron alignment requirements can be realized vertically through the use of deposited solder and vertical alignment features, as demonstrated in ref. ^21^. Correspondingly, after thorough calibration, state-of-the-art flip-chip bonding and transfer printing can be used to realize similar placement accuracies in terms of lateral alignment equipment, e.g., the 3*σ* of 1.5 µm reported with transfer printing^22^.

## Conclusion

We have demonstrated for the first time a compact hybrid laser architecture incorporating a PhC cavity reflector that is suitable for schemes where precise wavelength registration is required (e.g., dense WDM links in datacenter applications). This configuration leads to very high stored energy densities that are unprecedented in a PhC cavity, which make the lasing wavelength a sensitive function of the laser power through local heat generation in the resonator’s small modal volume due to nonlinear absorption in silicon. We have exploited this inherent passive mechanism (which we have named power tuning) to balance ambient temperature changes with controlled absorptive heating in the PhC. Using power tuning, a lasing wavelength stability of ±0.38 nm over a temperature range of 20–80 °C under uncooled operation was demonstrated. We envisage future silicon chips comprising arrays of PhC cavities with densely spaced wavelengths coupled to an array of standardized RSOAs, giving a dense grid of transmitters. This geometry can efficiently utilize available space, on the order of 100 × 500 μm^2^, for both silicon and III–V chips, with a demonstrated output power in the milliwatt range, thereby providing a route to high capacity interconnects in a datacenter environment by means of cooler-less dense WDM. Moreover, this laser platform has potential applications in optical sensing and in nonlinear optics.

The laser architecture proposed in this article is fully compatible with existing fabrication and integration technologies and is thus suitable for production of such devices at a large scale. The hybrid integration approach has been specifically selected here, as it enables independent optimization and fabrication of the active and passive regions while allowing cost-effective use of III–V materials^23^. This alleviates problems commonly encountered in wafer-bonded or heterogeneously integrated lasers, which arise from the difficulties in simultaneously realizing evanescent coupling, good thermal conductivity, and high gain. Further to that, both the silicon and the gain chips can be tested and screened prior to assembly to improve yields.

The PhC cavity-based reflector is compatible with low-loss waveguiding platforms, such as siloxane polymers^24^ and silicon nitride^25^, making the proposed laser devices suitable for integration with active and passive components previously demonstrated for the vertically coupled PhC platform^26–28^ toward the realization of more complex, power-efficient Si photonic systems.
